# Supplementary material for: NEUROD1 reinforces endocrine cell fate acquisition in pancreatic development
Source: Nat Commun. 2023 Sep 9;14:5554. doi: 10.1038/s41467-023-41306-6 (PMC10492842; doi:10.1038/s41467-023-41306-6)
Supplement: Supplementary file 1 — Supplementary Information [file 41467_2023_41306_MOESM1_ESM.pdf]

Supplementary information

NEUROD1 reinforces endocrine cell fate acquisition in pancreatic development

Romana Bohuslavova[1*], Valeria Fabriciova[1*], Ondrej Smolik[1*], Laura Lebrón-Mora[1], Pavel Abaffy[2], Sarka Benesova[2], Daniel Zucha[2], Lukas Valihrach[2], Zuzana Berkova[3], Frantisek Saudek[3], and Gabriela Pavlinkova[1]

[1]Laboratory of Molecular Pathogenetics, Institute of Biotechnology CAS, 25250 Vestec, Czechia

[2]Laboratory of Gene Expression, Institute of Biotechnology CAS, 25250 Vestec, Czechia

[3]Diabetes Centre, Institute for Clinical and Experimental Medicine, 14021 Prague, Czechia

Correspondence should be addressed to G.P. (email: gpavlinkova@ibt.cas.cz)

Table of contents

1. Supplementary Figures 1 to 5
2. Supplementary Tables 1 to 6

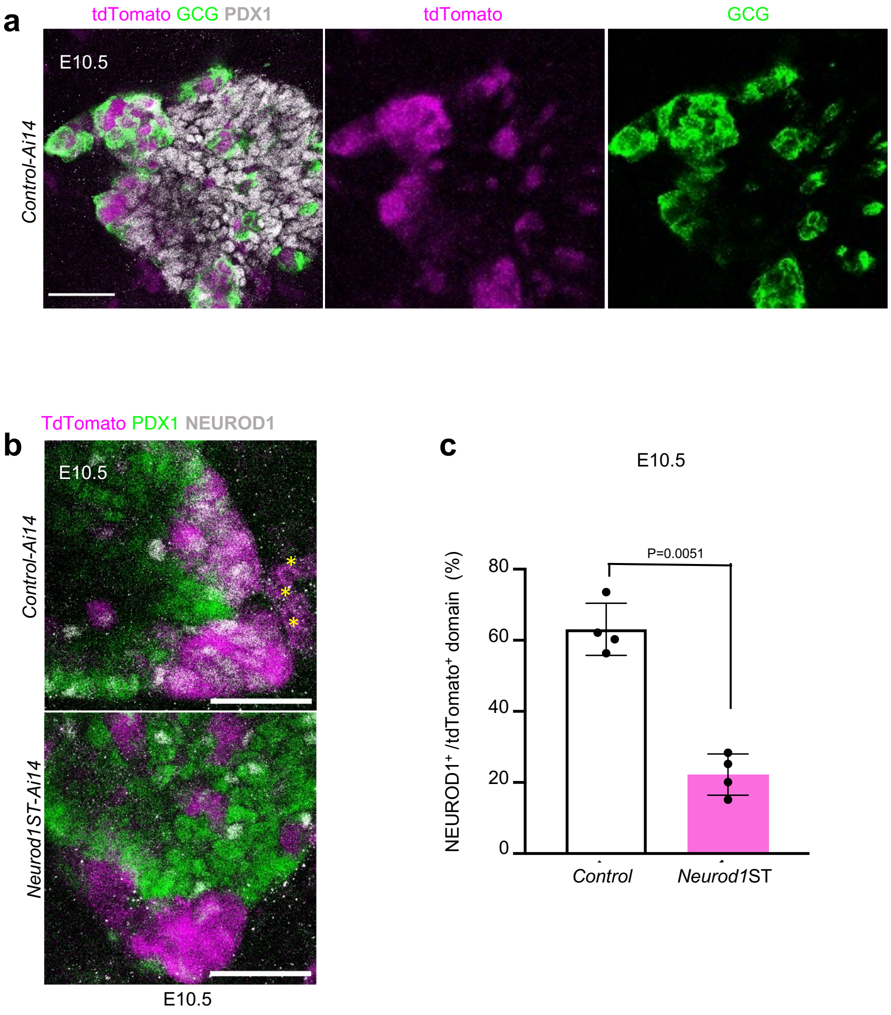

Supplementary Figure 1. Efficient deletion of NEUROD1 in the E10.5 pancreas of *Neurod1*ST. a Representative confocal images of early endocrine cell clusters co-expressing tdTomato and glucagon (GCG) in the E10.5 dorsal pancreatic bud of *Control-Ai14* embryos (genotype *Neurod1*$^{loxP/+}$; *Neurod1*$^{Cre/+}$; *Ai14*). The dorsal pancreatic bud is visualized by whole-mount immunohistochemistry staining of PDX1 (a marker of pancreatic multipotent progenitors). **b** A detail of the endocrine cell clusters with the co-expression of tdTomato and NEUROD1 in the E10.5 *Control-Ai14* and *Neurod1-Ai14* pancreas of whole-mount immunohistochemistry staining. *Neurod1ST-Ai14* pancreases at E10.5. Note, the reduction of NEUROD1 expression in tdTomato domain of *Neurod1ST-Ai14* (genotype *Neurod1*$^{loxP/loxP}$; *Neurod1*$^{Cre/+}$; *Ai14).*Asterisks indicate red blood cells. **c** Semi-quantitative measurements of NEUROD1 protein in the tdTomato^{+} domain of *Control* and *Neurod1*ST was performed using the thresholding tool of the ImageJ. Data are presented as mean ± SD (n = 4 pancreases/genotype); unpaired two-tailed *t*-test. Source data are provided as a Source Data file. Scale bars, 50 µm.

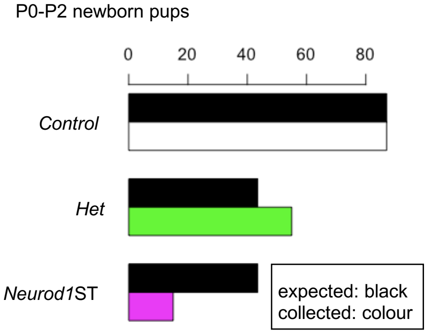

Supplementary Figure 2. Reduced survival of *Neurod1*ST. Bar plot presenting the distribution of genotypes of collected newborn pups at P0-P2. The expected Mendelian ratio is indicated (black). Source data are provided as a Source Data file.

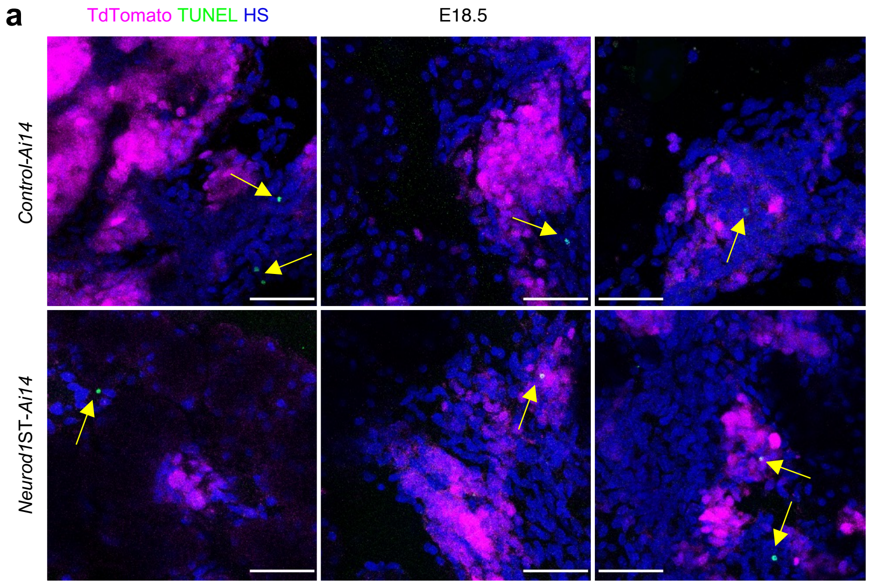

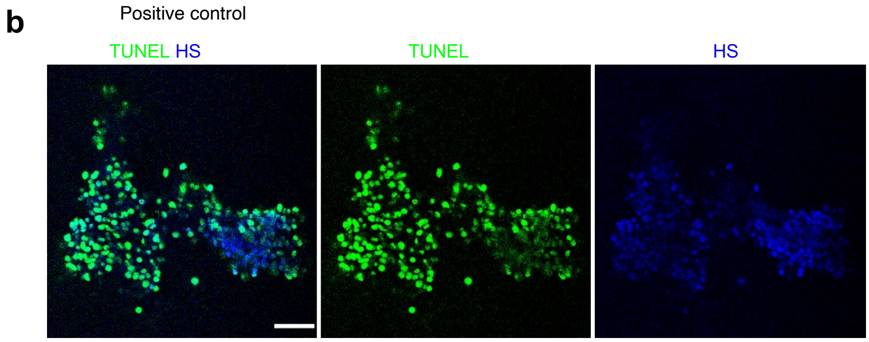

Supplementary Figure 3. TUNEL analysis shows a similar level of apoptosis in the control and mutant pancreas. a Representative images of apoptotic cells stained by TUNEL and tdTomato^{+} endocrine cells in the sections of pancreases at E18.5. Nuclei were stained using Hoechst (HS). Arrows indicate cells that were positive for TUNEL. **b** Positive control for TUNEL assay. Scale bars, 50 µm.

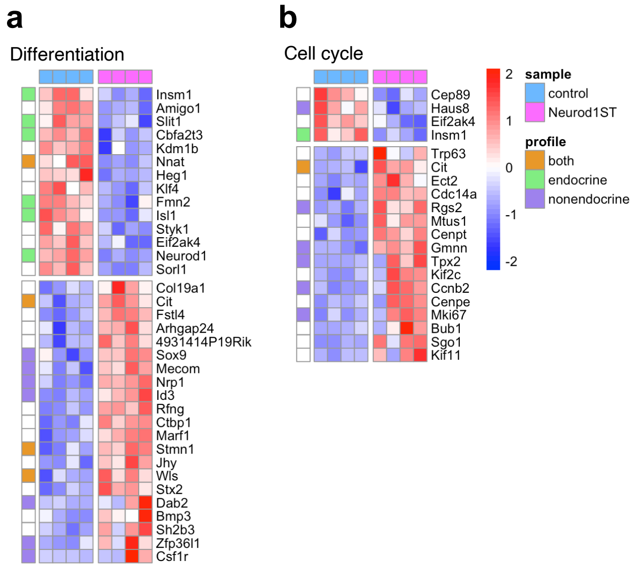

Supplementary Figure 4. Transcriptome changes in *Neurod1*ST based on bulk-cell RNA-seq. **a** Heatmap of differentially expressed genes within differentiation-related gene cluster assembled by DAVID tool. **b** Heatmap of differentially expressed genes within cell cycle-related gene cluster assembled by DAVID tool.

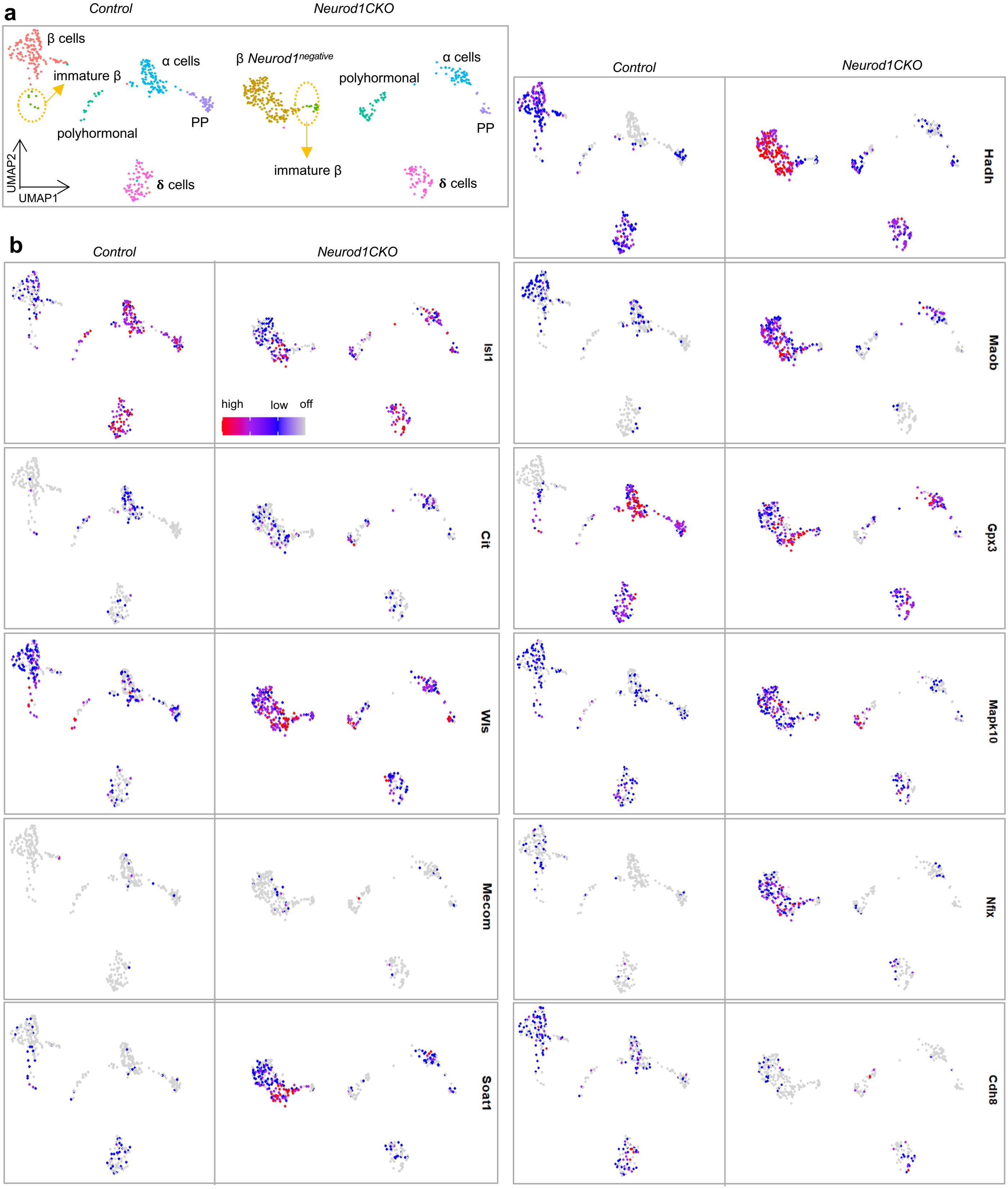

Supplementary Figure 5. Transcriptome changes in the adult *Neurod1CKO* endocrine cells based on single-cell RNAseq. (a) UMAP visualization (in two dimensions [UMAP_1 and UMAP_2]) of endocrine cells from the adult *Control* and *Neurod1CKO* pancreas (different colors mark distinct cell types annotated based on the expression of signature genes in Fig. 6; each dot represents one cell). Clusters corresponding to α, β, δ, PP (γ), immature β , β cells not expressing *Neurod1* (in *Neurod1CKO*), and polyhormonal cells are shown in cropped and transformed UMAP plots that excludes a cluster of β cells expressing *Neurod1* (cluster *Neurod1* ^{positive}) in *Neurod1CKO* plots for easier visualization. (**b**) Two-dimensional embedding and unsupervised clustering of endocrine profiles in UMAP visualizations of selected genes in *Control* and *Neurod1CKO*.

Supplementary Table 1.
Primers used for genotyping

gene	sequence
Cre_F	CCATTTTGCAGTGGACTCCT
Cre_R	ACGGACAGAAGCATTTTCCA
Neurod1_F	ACCATGCACTCTGTACGCATT
Neurod1_R	GAGAACTGAGACACTCATCTG

Supplementary Table 2.
Primers used for qPCR

gene	sequence
Cpe_F	AGCTACCTGGAGCAGATACAC
Cpe_R	TCGCCAGTAATCCCCATCCT
Gcg_F	CAGAAGAAGTCGCCATTGCC
Gcg_R	AAGTCCCTGGTGGCAAGATT
Ins1_F	GACCAGCTATAATCAGAGACCATC
Ins1_R	GTAGGAAGTGCACCAACAGG
Ins2_F	GGCTTCTTCTACACACCCAT
Ins2_R	CCAAGGTCTGAAGGTCACCT
Resp18_F	AGGATATGAAGGCAGTTTCCCC
Resp18_R	TTCACCTTGACTGTTGGACTCTTT
Scg5_F	GTTTGGGCAAGTGGAACAAGAA
Scg5_R	GAGAAGTGGGGGACAGATTTC
Slc2a5_F	GAATAAACTGGGCAGAAAAGGGG
Slc2a5_R	TAGGGACCACGTTGGAAGAGA
Smarcd2_F	CAACCATCTGGTGGAGTGGCAT
Smarcd2_R	AGGCGGGGGTCCAGTTTAT
Tox3_F	GGGTACTACGGCTACAGCAAG
Tox3_R	TGTGGAATGTCTGCTCACTGG

Supplementary Table 3.
Primary and secondary antibodies used for immunohistochemical staining

name	host	manufacturer	catalogue number	dilution
anti-alpha-amylase	rabbit	Sigma-Aldrich	A8273	1:2500
anti-C peptide 1	rabbit	Beta cell consortium	Ab657	1:1000
anti-C peptide 2	rabbit	Beta cell consortium	Ab1042	1:3000
anti-glucagon	mouse	Abcam	Ab10988	1:500
anti-glucagon	goat	Bio-Rad	4660-1140	1:500
anti-insulin	guinea pig	Abcam	Ab7842	1:50
anti-insulin	guinea pig	Genetex	GTX27842	1:50
anti-ISL1	mouse	Developmental Hybridoma bank	39.4D5	1:200
anti-Ki67	rabbit	Cell signalling	9129	1:400
anti-NEUROD1	rabbit	Abcam	Ab109224	1:500
anti-PDX1	rabbit	Abcam	Ab47267	1:2000
anti-PDX1	mouse	Developmental Hybridoma bank	F6A11	1:3
anti-SOX9	rabbit	Sigma-Aldrich	AB5535	1:1000
Alexa Fluor® 594-conjugated AffiniPure Anti-Rabbit IgG (H+L)	donkey	Jackson ImmunoResearch	711-585-152	1:500
Alexa Fluor® 488-conjugated AffiniPure Anti-Mouse IgG (H+L)	donkey	Jackson ImmunoResearch	715-545-150	1:500
Alexa Fluor® 647-conjugated AffiniPure Anti-Guinea pig IgG (H+L)	donkey	Jackson ImmunoResearch	706-605-148	1:500
Alexa Fluor® 647-conjugated AffiniPure Anti-Goat IgG (H+L)	donkey	Jackson ImmunoResearch	705-605-147	1:500
DyLight 405-conjugated AffiniPure Goat Anti-Guinea Pig IgG (H+L)	goat	Jackson ImmunoResearch	106-475-003	1:500
Alexa Fluor® 488 AffiniPure Goat Anti-Mouse IgG (H+L)	goat	Jackson ImmunoResearch	115-545-146	1:500
Alexa Fluor® 594 AffiniPure Goat Anti-Rabbit IgG (H+L)	goat	Jackson ImmunoResearch	111-585-144	1:500

Supplementary Table 4.
Primary antibodies used for LFSM

name	host	manufacturer	catalogue number	dilution
anti-GLP1	mouse	Abcam	Ab111125	1:1000
anti-insulin	guinea pig	Genetex	GTX27842	1:50

Supplementary Table 5.
Primary antibodies used for CUT&Tag profiling

name	host	manufacturer	catalogue number	dilution
anti-H3K4me3	rabbit	Active Motif	39159	1:100
anti-H3K27me3	rabbit	Active Motif	39155	1:100
IgG	rabbit	Active Motif	13-0047	0.5µg

Supplementary Table 6.
Scale factors size used in CUT&Tag profiling

track	PCR cycles	cell count	CPM factor	spike-in factor
H3K4me3 control	27	4000	1,009	14,49
H3K4me3 Neurod1ST	27	4000	0,991	14,55
H3K27me3 control	25	20605	0,925	25,37
H3K27me3 Neurod1ST	26	4000	1,081	107,01
IgG negative control	28	6370	0,780	NA
