# Supplementary material for: NEUROD1 reinforces endocrine cell fate acquisition in pancreatic development
Source: Nat Commun. 2023 Sep 9;14:5554. doi: 10.1038/s41467-023-41306-6 (PMC10492842; doi:10.1038/s41467-023-41306-6)
Supplement: Supplementary file 3 — Description of Additional Supplementary Files [file 41467_2023_41306_MOESM3_ESM.pdf]

Description of Additional Supplementary Files

File Name: Supplementary Data 1
Description: Analysis of differentially expressed genes between *Neurod1*ST mutant and control endocrine cells at E15.5 identified by bulk RNA-seq.

File Name: Supplementary Data 2
Description: Differentially expressed genes between control and *Neurod1CKO* mutant beta cells and alpha cells identified by scRNA-seq.

File Name: Supplementary Movie 1
Description: **E18.5 *Control* tdTomato GLP1 INS**. Microdissected pancreas of tdTomato reporter *Control-Ai14* mice was cleared (CUBIC protocol), immunolabeled, imaged, and reconstructed in 3D using light-sheet fluorescence microscopy (LFSM). Video shows the distribution and formation of islets in the anatomical microenvironment of the pancreas at E18.5; tdTomato$^+$ endocrine cell population (magenta), β cells with expression of insulin (white), and α cells expressing glucagon-like peptide-1 (GLP1, green).

File Name: Supplementary Movie 2
Description: **E18.5 *Neurod1*ST tdTomato GLP1 INS**. Microdissected pancreas of tdTomato reporter *Neurod1*ST-*Ai14* mice were cleared (CUBIC protocol), immunolabeled, imaged, and reconstructed in 3D using light-sheet fluorescence microscopy (LFSM). Video shows the distribution and formation of islets in the anatomical microenvironment of the pancreas; tdTomato$^+$ endocrine cell population (magenta), β cells with expression of insulin (white), and α cells expressing glucagon-like peptide-1 (GLP1, green).
