# Supplementary material for: NEUROD1 reinforces endocrine cell fate acquisition in pancreatic development
Source: Nat Commun. 2023 Sep 9;14:5554. doi: 10.1038/s41467-023-41306-6 (PMC10492842; doi:10.1038/s41467-023-41306-6)
Supplement: Supplementary file 8 — Reporting Summary [file 41467_2023_41306_MOESM8_ESM.pdf]

nature portfolio

Corresponding author(s): Gabriela Pavlinkova, NCOMMS-23-04110

Last updated by author(s): 2023- 08-04

Reporting Summary

Nature Portfolio wishes to improve the reproducibility of the work that we publish. This form provides structure for consistency and transparency in reporting. For further information on Nature Portfolio policies, see our Editorial Policies and the Editorial Policy Checklist.

Statistics

For all statistical analyses, confirm that the following items are present in the figure legend, table legend, main text, or Methods section.

n/a	Confirmed	
☐	☒	The exact sample size (*n*) for each experimental group/condition, given as a discrete number and unit of measurement
☐	☒	A statement on whether measurements were taken from distinct samples or whether the same sample was measured repeatedly
☐	☒	The statistical test(s) used AND whether they are one- or two-sided<br>*Only common tests should be described solely by name; describe more complex techniques in the Methods section.*
☒	☐	A description of all covariates tested
☒	☐	A description of any assumptions or corrections, such as tests of normality and adjustment for multiple comparisons
☐	☒	A full description of the statistical parameters including central tendency (e.g. means) or other basic estimates (e.g. regression coefficient) AND variation (e.g. standard deviation) or associated estimates of uncertainty (e.g. confidence intervals)
☐	☒	For null hypothesis testing, the test statistic (e.g. *F*, *t*, *r*) with confidence intervals, effect sizes, degrees of freedom and *P* value noted<br>*Give P values as exact values whenever suitable.*
☒	☐	For Bayesian analysis, information on the choice of priors and Markov chain Monte Carlo settings
☒	☐	For hierarchical and complex designs, identification of the appropriate level for tests and full reporting of outcomes
☒	☐	Estimates of effect sizes (e.g. Cohen's *d*, Pearson's *r*), indicating how they were calculated

Our web collection on statistics for biologists contains articles on many of the points above.

Software and code

Policy information about availability of computer code

Data collection	ZEN 2.1 Black; Leica Application Suite; NIS-Elements; FIJI; CFX384 Real-Time PCR Detection System (Bio-Rad); STAR (v2.7.0c); NextSeq System Suite v2.2.0, Zeiss Lightsheet Z.1 microscope, Illumina NovaSeq 6000 SP, MiSeq (Illumina).
Data analysis	3D Lightsheet imaging data were processed by IMARIS software v8.1.1. Statistical analyses were performed in GraphPad Prism 9. Raw sequencing data were analyzed in R using following list of packages: R STAR v597 2.7.0c, cutadapt v1.18, TrimmomaticPE v0.36, bowtie v1.2.2, Rsubread v2.0.1, DropletUtils R package, DESeq2 v1.26.0, Seurat R package, CibersortX, SEACR (ver. 1.3), STARsolo (STAR version 2.7.3a) and additional tools: HOMER (ver. 4.10), GOTermFinder v0.86, g: Profiler version e104_eg51_p15_3922dba.

For manuscripts utilizing custom algorithms or software that are central to the research but not yet described in published literature, software must be made available to editors and reviewers. We strongly encourage code deposition in a community repository (e.g. GitHub). See the Nature Portfolio guidelines for submitting code & software for further information.

Data

Policy information about availability of data

All manuscripts must include a data availability statement. This statement should provide the following information, where applicable:

- Accession codes, unique identifiers, or web links for publicly available datasets
- A description of any restrictions on data availability
- For clinical datasets or third party data, please ensure that the statement adheres to our policy

All data generated and/or analyzed during the current study are available within the paper Figure 1-7, and its Supplementary Figures 1-5, Supplementary Table 1-6 and 2, and Supplementary Data 1 and Data 2, Supplementary Movie S1-S4. The FACS-sorted neuron RNA sequencing data generated in this study have been deposited in the GEO database under accession code GSE182575 (https://www.ncbi.nlm.nih.gov/geo/query/acc.cgi?acc=GSE182575). Single-cell RNA-seq data will be deposited at the GEO database and accession number will be provided before publication. Source data are provided within this paper.

Research involving human participants, their data, or biological material

Policy information about studies with human participants or human data. See also policy information about sex, gender (identity/presentation), and sexual orientation and race, ethnicity and racism.

Reporting on sex and gender	*Use the terms sex (biological attribute) and gender (shaped by social and cultural circumstances) carefully in order to avoid confusing both terms. Indicate if findings apply to only one sex or gender; describe whether sex and gender were considered in study design; whether sex and/or gender was determined based on self-reporting or assigned and methods used. Provide in the source data disaggregated sex and gender data, where this information has been collected, and if consent has been obtained for sharing of individual-level data; provide overall numbers in this Reporting Summary. Please state if this information has not been collected. Report sex- and gender-based analyses where performed, justify reasons for lack of sex- and gender-based analysis.*
Reporting on race, ethnicity, or other socially relevant groupings	*Please specify the socially constructed or socially relevant categorization variable(s) used in your manuscript and explain why they were used. Please note that such variables should not be used as proxies for other socially constructed/relevant variables (for example, race or ethnicity should not be used as a proxy for socioeconomic status). Provide clear definitions of the relevant terms used, how they were provided (by the participants/respondents, the researchers, or third parties), and the method(s) used to classify people into the different categories (e.g. self-report, census or administrative data, social media data, etc.) Please provide details about how you controlled for confounding variables in your analyses.*
Population characteristics	*Describe the covariate-relevant population characteristics of the human research participants (e.g. age, genotypic information, past and current diagnosis and treatment categories). If you filled out the behavioural & social sciences study design questions and have nothing to add here, write "See above."*
Recruitment	*Describe how participants were recruited. Outline any potential self-selection bias or other biases that may be present and how these are likely to impact results.*
Ethics oversight	*Identify the organization(s) that approved the study protocol.*

Note that full information on the approval of the study protocol must also be provided in the manuscript.

Field-specific reporting

Please select the one below that is the best fit for your research. If you are not sure, read the appropriate sections before making your selection.

☒ Life sciences ☐ Behavioural & social sciences ☐ Ecological, evolutionary & environmental sciences

For a reference copy of the document with all sections, see nature.com/documents/nr-reporting-summary-flat.pdf

Life sciences study design

All studies must disclose on these points even when the disclosure is negative.

Sample size	Animal numbers were minimized to conform to ethical guidelines while accurately establishing the phenotype (morphological and cellular analyses) and performed the molecular analyses (bulk- and single-cell-RNA-seq, and Cut&Tag-seq).
Data exclusions	No sample was formally excluded from analyses.
Replication	When descriptive data are presented without quantification, the experiments were replicated at least three times (biological replicates) and all attempts at replication were successful. The exact sample size (n) for each experimental group is indicated in the text or figures for quantified parameters.
Randomization	Batches of animals from different litters/parents were used for experiments. The animals were then randomly allocated to each experimental group.

Blinding | Analyses were performed blind to the genotype.

Reporting for specific materials, systems and methods

We require information from authors about some types of materials, experimental systems and methods used in many studies. Here, indicate whether each material, system or method listed is relevant to your study. If you are not sure if a list item applies to your research, read the appropriate section before selecting a response.

Materials & experimental systems

n/a	Involved in the study
☐	☒ Antibodies
☒	☐ Eukaryotic cell lines
☒	☐ Palaeontology and archaeology
☐	☒ Animals and other organisms
☒	☐ Clinical data
☒	☐ Dual use research of concern
☒	☐ Plants

Methods

n/a	Involved in the study
☒	☐ ChIP-seq
☐	☒ Flow cytometry
☒	☐ MRI-based neuroimaging

Antibodies

Antibodies used | All antibodies used in the study are listed in Supplementary Table 3-5. The description includes supplier name, host, catalog number, and used dilution.

Validation | Each antibody was validated for the species (mouse) and application (immunohistochemistry) by the correspondent manufacturer or/and within the lab.

Animals and other research organisms

Policy information about studies involving animals; ARRIVE guidelines recommended for reporting animal research, and Sex and Gender in Research

Laboratory animals | Neurod1Cre transgenic mice (Tg(Neurod1-cre)1Able/J, # 028364, Jackson Laboratory); Cre-reporter tdTomato line (TomatoAi14, B6.Cg-Gt(ROSA)26Sortm14(CAG-tdTomato)Hze, # 7914 Jackson Laboratory); Isl1Cre transgenic mice (Isl1tm1(cre)Sev/J, # #024242 Jackson Laboratory); and Neurod1loxP/loxP mice gift from Prof. Bernd Fritzsch, Iowa University, USA. Both males and females were used for experiments, and mice were randomly allocated to experimental groups. Lines are a mixed C57BL/6/sv129 background. The mice were housed in 12-hour light/dark cycles and were fed ad libitum.

Wild animals | No wild animals were used in this study.

Reporting on sex | Both males and females were used for experiments, no differences in survival or blood glucose levels were detected between males and females.

Field-collected samples | No field-collected samples were used in this study.

Ethics oversight | All procedures performed on mice were carried out in accordance with the ethical guidelines of the Care and Use of Laboratory Animals (National Research Council. Washington, DC. The National Academies Press, 1996). The design of experiments was approved by the Animal Care and Use Committee of the Institute of Molecular Genetics, Czech Academy of Sciences (protocol # 878/2022).

Note that full information on the approval of the study protocol must also be provided in the manuscript.

Flow Cytometry

Plots

Confirm that:

☒ The axis labels state the marker and fluorochrome used (e.g. CD4-FITC).

☒ The axis scales are clearly visible. Include numbers along axes only for bottom left plot of group (a 'group' is an analysis of identical markers).

☒ All plots are contour plots with outliers or pseudocolor plots.

☒ A numerical value for number of cells or percentage (with statistics) is provided.

Methodology

Sample preparation	Cre-reporter tdTomato line (TomatoAi14) was used to genetically label endocrine cells. Fluorescence-activated cell sorted cells were prepared from embryos with the genotypes Neurod1-Cre, Neurod1loxP/loxP, TomatoAi14; and Neurod1-Cre, Neurod1loxP/+, TomatoAi14. Briefly, microdissected pancreases from E15.5 embryos trypsinized by lysis solution (0.05% trypsin, 0.53mM EDTA Dulbecco's PBS). The lysis was stopped by adding 600 µl of FACS buffer (Dulbecco's PBS, 10mM EGTA, and 2% FBS). After spinning down the samples, the supernatant was removed, and cell pellets were resuspended in 500 µl of ice-cold FACS buffer. Immediately before sorting, cells were passed through a 50 µm cell sieve (CellTricsTM, Sysmex Amercica Inc.) into a sterile 5 ml polystyrene round-bottom falcon to remove clusters of cells and kept on ice. TdTomato+ cells were sorted using a flow cytometer BD FACSAriaTM Fusion through a 100 µm nozzle in 20 psi. 100 sorted cells from individual samples, containing dissociated cells were collected into individual wells of 96-well plate containing 5 µl of lysis buffer of NEB Next single-cell low input RNA library prep kit for Illumina. Plates were frozen immediately on dry ice and stored at −80°C. For CUT&Tag profiling study, ~5000-20000 tdTomato+ cells per sample were sorted and immediately used.
Instrument	BD FACSAriaTM Fusion
Software	BD FACSDiva™ Software
Cell population abundance	Cells were sorted directly into lysis buffer, as such the purity of the cell population was not confirmed by post-sorting. Purity was instead confirmed through RNAseq by cross validation with known cell-type markers. During the optimalization of the protocol, post-sorted cells were initially checked for presence of the fluorescent reporter. Purities of grater than 95% were routinely observed.
Gating strategy	Gating strategy were established based on the resort of positive population. Negative population was also resorted to confirm the efficiency of gating strategy.

☐ Tick this box to confirm that a figure exemplifying the gating strategy is provided in the Supplementary Information.
